# Angiotensin-Converting Enzyme and Hypertension: A Systemic Analysis of Various ACE Inhibitors, Their Side Effects, and Bioactive Peptides as a Putative Therapy for Hypertension

**DOI:** 10.1155/2023/7890188

**Published:** 2023-06-21

**Authors:** Hafiz Ahmad, Huma Khan, Shabirul Haque, Shameem Ahmad, Namita Srivastava, Azhar Khan

**Affiliations:** ^1^RAK College of Medical Sciences, RAK Medical & Health Sciences University, Ras al Khaimah, UAE; ^2^Microbiology and Molecular Division-RAK Hospital, Ras al Khaimah, UAE; ^3^Faculty of Biotechnology and Applied Sciences, Shoolini University of Biotechnology and Management Sciences, Solan, India; ^4^The Feinstein Institute of Medical Research, Northwell Health, Manhasset, NY, USA; ^5^Department of Orthopedics, Lady Hardinge Medical College, New Delhi, India

## Abstract

Hypertension is a major risk factor for heart attack, produce atherosclerosis (hardening of the arteries), congestive heart failure, stroke, kidney infection, blindness, end-stage renal infection, and cardiovascular diseases. Many mechanisms are involved in causing hypertension, i.e., via calcium channels, alpha and beta receptors, and the renin-angiotensin system (RAS). RAS has an important role in blood pressure control and is also involved in the metabolism of glucose, homeostasis, and balance of electrolytes in the body. The components of RAS that are involved in the regulation of blood pressure are angiotensinogen, Ang I (angiotensin I), Ang II (angiotensin II), ACE (angiotensin-converting enzyme), and ACE 2 (angiotensin-converting enzyme 2). These components provide for relevant therapeutic targets for the treatment of hypertension, and various drugs are commercially available that target individual components of RAS. Angiotensin receptor blockers (ARBs) and ACE inhibitors are the most popular among these drugs. ACE is chosen in this review as it makes an important target for blood pressure control because it converts Ang I into Ang II and also acts on the vasodilator, bradykinin, to degrade it into inactive peptides. This review highlights various aspects of blood pressure regulation in the body with a focus on ACE, drugs targeting the components involved in regulation, their associated side effects, and a need to shift to alternative therapy for putative hypertension treatment in the form of bioactive peptides from food.

## 1. Introduction

Hypertension or high blood pressure, generally called arterial hypertension, is defined by the American Heart Association as persistent high blood pressure consistently higher or above 140/90 mm Hg with systolic 130 mm Hg or higher and diastolic 80 mm Hg or higher affecting billions of people and is a common factor of death [1]. Hypertension is assessed based on many factors including genetic factors and hereditary factors and in most cases can also be attributed to obesity, diabetes, and depression [2]. Hypertension is also recognized as the permanent increase in systolic blood pressure in the arterial blood vessels [3]. Elevated pressure on the walls of blood vessels relies on several factors: pumping of the heart, accumulation of fluid, and the thickness of the vascular walls [4]. As an outcome of high blood pressure, the elasticity of the arteries is reduced, and also the accumulation of cholesterol in the walls occurs resulting in the blockage of vessels which is the *raison d'etre* for the organ damage that is caused as a result of hypertension. Hypertension accounts for 30–45% of the general population of which 60% are in the 60-65 years age group [5]. The World Health Organization (WHO) considers hypertension a significant preventable cause of worldwide death [6]. Hypertension is associated with an increased risk of cardiovascular pathologies and mortality in all age groups [7]. To treat high levels of blood pressure, an arsenal of antihypertensive drugs is available: calcium channel blockers, diuretics, beta blockers, ARBs, and ACE inhibitors [8, 9]. Some Indian studies showed an increase in the occurrence of hypertension about thirty times in the urban population in the 55-year-age group and about ten times in the rural population [6]. An important consideration of any systolic blood pressure analysis is whether the disorder is “primary” or “secondary.” Primary or secondary classifications for systolic blood pressure imply to the cause of high blood pressure and have nothing to do with the seriousness of the condition [10]. Primary or essential hypertension involves a rise in blood pressure without any known cause or medical condition, and some of the associated causes are lack of regular exercise, sedentary lifestyle, poor diet, and hereditary factors. Secondary hypertension, on the other hand, has a medical condition associated with it such as conditions affecting certain organs like the kidneys, endocrine system, and heart. It can be suspected in patients with an established history of familial hypertension and in women with pregnancy.

## 2. Mechanisms Involved in Hypertension

Different targets for regulating hypertension in the body include the renin-angiotensin system, calcium channels, and beta and *α*1-adrenergic receptors [11–13]. Calcium channel blockers (CCBs) are mainly utilized as antihypertensive drug targets. CCBs work by inhibiting the calcium ion flow into vascular smooth muscles, thereby dilating the arteries [13]. These blockers are mainly N-type, L-type, and T-type. Voltage-dependent calcium channels are present in the zona glomerulosa of the human adrenal gland. The drugs acting on Ca^+^ channels are listed (Table 1).

These inhibitors have some side effects like constipation and dizziness. CCBs, apart from lowering blood pressure, also promote fibrinolysis by increasing the levels of tissue plasminogen activator (t-PA), decreasing inflammatory markers, inhibiting muscle proliferation, extracellular matrix formation, and inhibition of apoptosis of endothelial cells [19–22]. Amlodipine, a calcium channel blocker, also increases the levels of the vasodilatory agent, nitric oxide (NO), and restores the stability between Ang II and NO, thereby reducing hypertension [23]. Beta blockers include a number of drugs that are disparate in pharmacodynamic and pharmacokinetic properties and have shown tremendous success in reducing mortality and cardiac arrest when targeting populations with heart failure or myocardial infarction. Beta blockers can therefore be used as a primary medicine for hypertension [24]. However, the utilization of *β*-blockers as the first line of medicine is controversial as no convincing results are yet available [25]. The *β*-blockers were not as successful in preventing heart attacks, strokes, and deaths as other available drugs such as diuretics, CCBs, and RAS inhibitors [22, 26, 27]. Beta-blockers commercially available are listed (Table 2) [16].

RAS is a central target for renal and cardiovascular defense and electrolyte balance [32]. Hormonal flow regulates blood quantity and arterial pressure to sustain sufficient organ perfusion. RAS begins with the release of renin into the circulation from the juxtaglomerular cells of the kidney. Renin converts angiotensinogen to a decapeptide, Ang I (Asp^1^-Arg^2^-Val^3^-Tyr^4^-Ile^5^-His^6^-Pro^7^-Phe^8^-His^9^-Leu^10^); this product is then cleaved by circulating and locally articulated ACE to an octapeptide, Ang II (Asp^1^-Arg^2^-Val^3^-Tyr^4^-Ile^5^-His^6^-Pro^7^-Phe^8^) [33, 34]. The predominant hormonal effects of Ang II are exerted by its binding to the Ang II type 1 (AT 1) and Ang II type 2 (AT 2) receptors [35]. Antihypertensive drugs that block RAS via diverse mechanisms include ACE inhibitors, direct renin inhibitors, and ARBs [36]. ARBs are an efficient group of antihypertensive agents and include drugs listed in Table 1 along with drugs used as ACE inhibitors [37]. The most widely used inhibitor is aliskerin for the curing of hypertension [38] and have demonstrated properties beyond blood pressure control [39].

Evidence suggests that chronic administration of ARBs to mice causes a significant reduction in vascular tone by activating eNOS-derived NO, even at doses below those that lower blood pressure. This effect was not seen after similar ACE inhibition or in eNOS-null animals. Telmisartan, an acute ex vivo pleiotropic ARB with AT1R-independent signalling properties, also inhibits aortic contractility in eNOS-dependent ways that are unrelated to traditional Ang II/AT1R signalling [40].

## 3. Angiotensin-Converting Enzyme (ACE)

The angiotensin-converting enzyme commission number is 3.4.15.1. ACE expression is found in the capillaries of the lungs and also in the endothelium of the kidney. It is necessary for the regulation of blood pressure and electrolyte homeostasis throughout the renin-angiotensin system [41]. RAS is the central monitor of arterial blood pressure, and ACE is one of its main regulators whose role is to convert the decapeptide angiotensin to an octapeptide angiotensin II [42], Ang-(1-9) to Ang-(1-7), and then other degrade its peptide to the inactive Ang-(1-5). ACE (kininase II) inactivates the vasodilators bradykinin-(1-9) and the kallikrein-kinin arrangement, by cleaving the C-terminal dipeptides. ACE ultimately cleaves its major metabolite bradykinin-(1-7) into the smaller fragment bradykinin-(1-5) [43]. Angiotensin-converting enzyme is recognized by several names: dipeptidyl carboxypeptidase I, kininase II, peptidase P, peptidyl-dipeptidase A, dipeptide hydrolase, peptidyl dipeptidase, and carboxycathepsin, based on its mode of action. The synthetic drugs for targeting ACE are mentioned above and are known as ACE inhibitors.

ACE inhibitors are usually administered orally, although intravenous formulations are available. The most common suffix for these medications is “-pril.” Some of the examples are lisinopril, ramipril, and captopril. According to the chemical composition, it is divided into three groups: sulfhydryl-containing ACE inhibitor (captopril), phosphorus-containing ACE inhibitor (fosinopril), and dicarboxylic-containing ACE inhibitors (benazepril, enalapril, lisinopril, moexipril, perindopril, quinapril, ramipril, and trandolapril) [44]. ACE inhibitors are a class of medicines that are used for the treatment of distinct disorders and are the first-class alternative for cardiovascular disease treatment [45]. ACE inhibitors do not depend on blood sugar levels and hence are the first choice for diabetic patients suffering from hypertension. ACE inhibition is the most recent advancement in the treatment of hypertension, and in the last recent years, it has become increasingly admired as the primary variety in the pharmacotherapy of this disease. As with additional classes of antihypertensive drugs, such as thiazide diuretics and 3-adrenergic blockers, the pharmacodynamics of this form of treatment has been most widely investigated using the first mediator of this class that became presented, captopril [46]. Some ACE inhibitor medicines are further part of a generic tablet by all of a calcium-channel blocker therapy or ‘water tablet' (diuretic) medicine. The main side effect associated with ACE inhibitors is low blood pressure (hypotension). Some uncommon side effects of ACE inhibitors include dry cough, inflammation of the eyes, throat, or tongue (angioedema), hives, irregular heartbeats (may be due to excessive potassium), dizziness or fainting, headache, and a decline in kidney function [47].

Inhibition of the ACE leads to bradykinin accumulation in the lung [47]. In human lung cancer tissue, bradykinin receptors [48–50] release vascular endothelial growth factor, which promotes angiogenesis [51] and matrix metalloproteinases [50, 52]. ACEIs also cause accumulation of the substance P (SP)/neurokinin (NK)-1 receptor, which is linked to tumour proliferation and angiogenesis [52, 53].

On the basis of decades of clinical observations on the use of ACEIs, a number of adverse effects have gained increasing attention. Interestingly, respiratory side effects have been a problem for a long time when ACEIs are used. A dry cough is a major side effect of respiratory side effects [54, 55]. Some studies have shown that taking ACEIs increases the risk of airway obstruction symptoms and makes the risk of bronchial asthma or asthma worse [56, 57]. Animal studies on carcinogenicity have shown negative results, but some studies have found that ACEIs are involved in angiogenesis, cell proliferation, and the growth of tumours [58, 59]. Recent research suggests that patients taking ACEIs may develop lung cancer [60–62]. ACEI inhibited tumour growth and metastasis in mice [63], according to various studies. As a result, the data on lung cancer and ACEIs are contradictory.

Two predominant forms of ACE are articulated in humans; these are somatic form and germinal form. The somatic form is abundantly found on the endothelial surface of lung vessels and other cell types and also on the surface of monocytes, smooth muscle cells, T lymphocytes, and adipocytes, whereas the germinal form is present exclusively in the testis. Both forms of ACE are present on the outer cell surface as ectoenzymes, where they hydrolyze circulating peptides. The soluble form of ACE, different from the membrane-bound form, is formed due to the ACE secretase action and is found in serum and other body fluids. Testicular ACE is the ancestral form of the molecule with a particular active site; its crystal structure was published in 2003 [41]. Somatic ACE arose as an implication of gene duplication and contains two active sites (Terminal N- and C-domains). The polypeptide chain of somatic ACE is 1,277 amino acids, whereas the testicular ACE is smaller with 701 amino acid residues [63].

## 4. Somatic ACE

Somatic ACE (sACE) has a significant role in the blood pressure regulatory mechanism (renin-angiotensin system). In somatic ACE, the polypeptide chain is 1,277 amino acids in length [63]. It is involved in the Ang II formation which is a potent vasoconstrictor and also degrades the vasodilator bradykinin, thereby resulting in increased blood pressure [64]. The activity of somatic ACE is highly dependent on the chloride ion concentration and is immobile in its deficiency, whereas the N domain is completely active at relatively low concentrations or even in the absence of chloride ions [65]. Somatic ACE has two diverse domains C and D, and each contains an observant active site [41]. Both of these domains are involved in Ang I formation and bradykinin degradation; therefore, both of these domains are important in systolic blood pressure regulation and cardiac stroke.

## 5. Testicular/Germinal ACE

Testicular ACE has an important role in reproduction and is 701 amino acids in length [63]. It has the same gene sequence as somatic ACE but has a tissue-specific promoter located within intron 12 [66]. Germinal ACE relies on chloride to a slight extent as compared to the C domain of sACE [67]. As previously observed, sACE has two active sites, whereas testicular ACE has only one active site [68].

## 6. Angiotensin-Converting Enzyme 2

**Figure 1:**
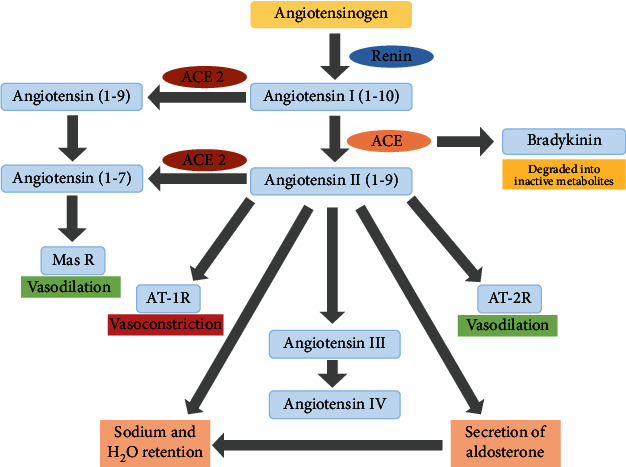
Schematic representation of the renin angiotensin aldosterone system. Abbreviations: Mas R: Mas Receptor; AT-1 R: angiotensin type 1 receptor; AT-2 R: angiotensin type 2 receptor; ACE: angiotensin-converting enzyme.

ACE 2 is a zinc metalloenzyme and carboxypeptidase situated as an ectoenzyme on the surface of endothelial and disparate cells. It has a great homology with ACE and catalyzes the cleavage of angiotensin I directed toward angiotensin 1-9, and angiotensin II into the vasodilator angiotensin 1-7 as demonstrated schematically (Figure 1). Although the main substrate for ACE 2 is Ang II, it has other physiological substrates as well. In addition to this, ACE 2 also has functions other than catalysis which include improving of intestinal impartial amino acid transport and acting as a receptor for a crucial respiratory disease-causing SARS virus [69]. ACE 2 negatively regulates the renin-angiotensin system and catalyzes the conversion of angiotensin II to angiotensin 1-7 that has vasodilatory effects, thereby counterbalancing ACE activity. Increasing evidence suggests that its enzymatic activity has a protective role in cardiovascular diseases [70]. Upregulation of ACE 2 expression is usually recognized as a therapeutic approach in conditions like acute myocardial infarction [71], diabetes [72], hypertension [73], lung grudge [74], fertility [75], and fibrotic disorders [76]. ACE 2 has been mainly localized in tissues like the liver, intestines, and lungs [77] and also found to be present in the brain [78] where it acts as an inner regulator of cardiovascular function [79].

## 7. Polymorphisms in Renin-Angiotensin System

Numerous studies regarding polymorphisms in genes associated with RAS have been established. These polymorphisms have been found to be associated with various cardiovascular diseases [80]. Polymorphism is another extensive topic which is beyond the aspect of this review; hence, the focus here shall only be on polymorphism associated with ACE.

## 8. ACE Insertion/Deletion Polymorphism

Angiotensin converting enzyme is the main component of the renin-angiotensin system, and its insertion and deletion polymorphism is reported in intron 16 [81, 82]. Many researchers are working to study the involvement of genetic polymorphisms in ACE and its subsequent relation with hypertension [83]. ACE insertion and deletion polymorphism is associated with the occurrence of myocardial infarction, cardiovascular disease [84], left ventricular hypertrophy [85], microalbuminuria [86, 87], and pregnancy hypertensive disorders [88]. Furthermore, these polymorphisms are also associated with differences in blood pressure levels and organ damage, which is commonly reported in hypertensive patients [89].

## 9. Antihypertensive Drugs and Associated Side Effects

Commercially available antihypertensive drugs are given as a single drug or in combination of more than one drug so as to regulate high blood pressure. Although these drugs are very potent in controlling blood pressure in hypertensive patients, they have a number of side effects, some common and others rare. Some of these effects include low blood pressure or hypotension, kidney failure, liver failure, and decreased white blood cell count. These antihypertensives may also lead to birth defects in pregnant women and therefore should not be consumed during pregnancy. Studies have been conducted on the effects of antihypertensive drugs apart from their blood pressure-lowering effects.

A study carried out by Schunkert et al. showed that patients consuming antihypertensive like diuretics, beta blockers, ACE inhibitors, and ARBs are at a high risk of gout. Beta blockers and diuretics also increase the serum levels of uric acid which is in turn associated with gout [90]. Another report by Choi et al. reported that there are experimental and epidemiological findings which are suggestive of the fact that there is a link between antihypertensive drug-induced photosensitivity and skin cancer that may be due to DNA damage induced in predisposed individuals [91]. Another study reported the association of various antihypertensive drugs with the risk of cancer, for example, long-term use of angiotensin receptor blockers was found to be linked to malignant melanoma and long-term use of diuretics was linked to squamous cell carcinoma [92].

## 10. Alternate/Complimentary Treatment for Hypertension

Drugs extensively used for the treatment of hypertension cause a plethora of side effects, some rare and others serious, as mentioned in the above section. The need therefore arises to consider other sources of therapy for hypertension in the form of herbal medicines that have few or no known side effects [85] and are natural, safe, and effective [93]. There are studies suggesting the efficacy of various natural and herbal sources in controlling hypertension which include medicinal plants that are utilized for their antihypertensive properties [93] and include *Allium sativum* (garlic), *Andrographis paniculata* (king of bitter), *Apium graveolens* (celery), *Bidens pilosa* L. (beggar's tick, black-jack, etc.), *Camellia sinensis* (tea), *Coptis chinensis* (goldthread), *Coriandrum sativum* (cilantro or coriander), *Crataegus* spp. (hawthorns), *Crocus sativus* (saffron), *Cymbopogon citratus* (lemongrass), *Hibiscus sabdariffa* (roselle), *Nigella sativa* (black cumin; seed of blessing), *Panax* (ginseng), *Salviae miltiorrhizae* (chinese sage), and *Zingiber officinale* (ginger) [94]. A study also reports the use of *Stevia* leaf for treating diabetes and hypertension [95]. These antihypertensive plants have a variety of phytochemicals that have protective effects against hypertension [96].

Apart from the antihypertensive plants mentioned above, honey has also been shown to have therapeutic effects on cardiovascular diseases. People have been using honey since time immemorial for treating a plethora of ailments, and researchers have just started reconsidering its role in nutrition and therapeutics [97]. The high content of flavonoids as well as phenolic acids in natural honey is of significance for human health including protection against cardiovascular diseases [98]. A study on animal models using Malaysian honey has shown beneficial effects in protecting against hypertension [99]. The use of honey has various limitations including contamination with pesticides and antibiotics; therefore, these limitations need to be considered before using it for therapeutic purposes, although there is significant evidence that proves its health benefits [100].

## 11. Bioactive Peptides from Natural Sources as ACE Inhibitors

Bioactive peptides that are derived from food are compounds originating from animals and plants that are produced by food processing and fermentation [31]. Apart from nutrition, these have other regulatory functions in the human system including antimicrobial, antidiabetic, antiinflammatory, immune-modulatory, antioxidative, and renin and ACE 1 inhibitory bioactivities. Some of the food products that act as sources of ACE-I inhibitory peptides include dairy products such as fermented cheese and yogurt, collagen from fish skins, soy, meat by-products, hemp seed, broccoli, traditional medicines of Chinese and Iranian origin, cereals, micro and macroalgae, sardine, sesame, corn milk, and eggs [32]. These food products are under the “Food for Specified Health Uses” regulatory framework [32].

The length of inhibitory peptides is usually short, about 12 amino acids in length. This short length has its advantages for the inhibitory activity of the peptide such as helping in absorption and thereby ensuring its bioavailability. Some studies have reported that the presence of certain conditions enhance the ACE binding ability of the peptides. These include the presence of certain amino acids (hydrophobic) at the C-terminal along with the presence of proline, leucine, tryptophan, phenylalanine, and tyrosine at the C-terminal of the peptide. ACE inhibitory bioactive peptides have three classifications, namely, inhibitor type, substrate type, and prodrug type on the basis of their mode of action [32]. In the case of inhibitory type peptide, no cleavage of the peptide occurs, and hence its activity remains unchanged. The substrate-type peptide, on the other hand, is cleaved by ACE, and hence its activity decreases gradually. In the prodrug type, the larger peptide undergoes cleavage into smaller potent ACE inhibitory peptides by ACE itself [32]. Numerous clinical studies have suggested that food nutrients like proteins, carbohydrates, and fats have a significant role in blood pressure regulation. Substitution of carbohydrates with proteins and monounsaturated fats has a positive effect on maintaining blood pressure and lowering the risk of heart diseases. This positive effect of protein intake is associated with the presence of bioactive peptide fragments that are known to have antihypertensive effects. ACE-inhibiting antihypertensive peptides were first isolated from snake venom, and this led to further research to isolate such peptides from food in the hope to find an alternative to traditional antihypertensive medicines [31]. A list of some of the food-derived antihypertensive peptides is given in Table 2 having ACE inhibitory effects in both animal models and human subjects.

An interesting study on human subjects showed that oral administration of milk-derived peptides, IPP and VPP, significantly reduced blood pressure levels in individuals of Finnish and Japanese racial origin, but the same peptides failed to do so in individuals of Danish and Dutch racial origin. When these studies are performed in animal models, the animals usually belong to a specific strain, whereas human subjects may be of different racial and genetic origin. This leads to discrepancies in the results and efficacy of bioactive peptides. Therefore, this creates a need for more and more clinical trials on human subjects of varied ethnicities to establish a solid ground for the antihypertensive nature of bioactive peptides [31].

## 12. Conclusion

The present review highlights the role of the angiotensin-converting enzyme in blood pressure regulation and how its inhibitors are used in the control of hypertension. From our review, we can safely conclude that commercially available synthetic ACE inhibitors pose numerous side effects in patients, and since hypertension is a chronic disease and leads to various cardiovascular disorders, the long-term consumption of these synthetic inhibitors can be harmful.

Therefore, based on evidences from existing literature, it can be concluded that natural remedies in the form of medicinal plants, bioactive peptides from food, and honey can be used as putative treatment option for hypertension and other cardiovascular diseases. Further research is definitely required in this direction to accept or reject these remedies for their antihypertensive nature.

## Figures and Tables

**Table 1:** Commercially available drugs and their respective classes.

S. no.	Class of drug	Commercial names	References
1.	Ang II type 1 (AT 1) receptor antagonists	Candesartan, losartan, telmisartan, eprosartan, valsartan, olmesartan, irbesartan	[14]
2.	Calcium channel blockers	Clevidipine, isradipine, amlodipine, aranidipine, benidipine, efonidipine, nimodipine, paradipine	[15]
3.	Beta blockers	Atenolol, betaxolol, bisoprolol, metoprolol, nadolol, propranolol, timolol	[16]
4.	ACE inhibitors	Captopril, moexipril, perindopril, ramipril, benzapril, fosinopril, lisinopril, trandolapril, quinapril	[17]
5.	Renin inhibitors	Aliskiren	[18]

**Table 2:** List of various bioactive peptides and their sources.

Source	Peptide/s	Reference
Whey	IW, VYPFPG, IPA, FP, VYP	[28, 29]
Milk	IPP, VPP	[28, 30, 31]
Rice	TQVY	[28]
Dried Bunito	LKPNM, LKP, IKP, IWH, IW, LYP	[29–31]
*α*_S_ 1-Casein	FFVAPFPGVFGK, IAK, YAKPVA, WQVLPNAVPAK	[30]
Bovine casein	MKP, GPL, GPV	[29, 30]
Egg	YPI, IRW, RADHPFL, YAEERYPIL	[29, 30]
Soybean	VLIVP, IFL, WL	[29]
Royal jelly	IY, VY, IVY	[29]
Mushroom	GEP	[29]
Canola	VSV, FL	[29]
Alaska Pollack	FGASTRGA	[29]
Chicken	GFXGTXGLXGF (X-hydroxyproline)	[29]
Spinach	MRWRD, MRW, LRIPVA, IAYKPAG	[29]
Wheat germ	IVY	[29]
Mung bean	KDYRL, VTPALR, KLPAGTLF	[29]
Marine shrimp	FCVLRP, IFVPAF, KPPETV	[29]
